# The mean platelet volume and plateletcrit as predictors of short-term outcome of acute ischemic stroke

**DOI:** 10.1186/s41983-018-0035-x

**Published:** 2019-01-08

**Authors:** Al-Amir Bassiouny Mohamed, Hassan Mohamed Elnady, Hazem Kamal Alhewaig, Hesham Moslem Hefny, Ashraf Khodery

**Affiliations:** 1 Department of Neurology and Psychological Medicine, Faculty of Medicine, Sohag University Hospital, Sohag, Egypt; 2 Department of Clinical Pathology, Faculty of Medicine, Sohag University Hospital, Sohag, Egypt

**Keywords:** Mean platelet volume, Plateletcrit, Outcome, Stroke

## Abstract

**Background:**

Activation of the platelet plays an important role in the process of atherosclerosis. Mean platelet volume (MPV) is significantly associated with the poor outcome of acute ischemic stroke while the results of studies about the relationship between plateletcrit (PCT) and stroke outcome were inconsistent. The aim of this work is to determine whether an association exists between MPV and plateletcrit (PCT) and outcome of acute ischemic stroke.

**Methods:**

We examined 157 patients with ischemic stroke, admitted to the Sohag University Hospital. The diagnosis of stroke was performed clinically according to The World Health Organization and confirmed by brain CT and MRI when needed. Platelet indices including MPV and PCT were assessed immediately (within 2 h) after admission. After 3 months, the functional outcome was assessed using the modified Rankin Scale (mRS) with assessment of the relationship between platelet indices and stroke outcome.

**Results:**

About 50% of the participants have favorable outcome. MPV was significantly higher in the unfavorable group (10.4 ± 2.3 fL) than in the favorable one (8.7 ± 1.3 fL) (*P* < 0. 001). MPV was an independent predictor of poor short-term outcome of acute stroke after controlling for confounders like diabetes mellitus. The mean PCT was significantly higher in the unfavorable group (0.28 ± 0.1%) than in the favorable one (0.25 ± 0.1%) (*P* = 0. 04) but not considered as an independent predictor of poor short-term outcome of acute stroke.

**Conclusions:**

MPV and PCT were significantly correlated with poor functional outcome, only MPV was an independent predictor of poor short-term outcome of acute stroke after controlling for confounders like DM, and these platelet indices can be used as a prognostic tool.

## Introduction

Under some pathological conditions like diabetes mellitus, obesity, metabolic syndrome, acute myocardial infarction (AMI), and stroke, the platelets may be larger in size and more reactive and this phenomenon seems to play an important role in several vascular diseases. Mean platelet volume (MPV) could be obtained from a simple and routine blood count and has a prognostic significance in stroke and AMI [1].

Platelet activation plays a key part in the process of atherosclerosis and then its potentially major adverse clinical outcomes, such as ischemic stroke and myocardial infarction (MI) [2–4]. It is well documented that there is a direct relationship between the platelet physiology and ischemic stroke by a number of observations, including the appearance of platelet thrombi on atheroma, and certain antiplatelet agents (e.g., aspirin) significantly reduce the incidence of ischemic stroke after initial transient ischemic attacks [5–8], and so measuring the platelet function by platelet indices (MPV and PCT) is useful in various clinical situations.

Many existing studies investigate the relationships between MPV and prognosis of coronary diseases [8–10], but studies between platelet indices and cerebrovascular accidents are lacking. Some studies found that increased MPV is associated with different stroke subtypes [8, 11, 12] while another study reported that MPV has been shown to be predictive of stroke recurrence [13].

PCT (also called platelet mass) is the volume occupied by platelets in the blood as a percentage and calculated according to the formula (PCT = platelet count × MPV/10,000). The number of platelets in the blood is maintained in an equilibrium state by regeneration and elimination [14–16].

Under physiologic conditions, the rate of platelet production is continuously regulated so that the circulating platelet mass (platelet count X MPV) is kept constant, and both MPV and platelet count are inversely associated [17, 18].

Platelet mass can be derived from data obtained from the routine full blood count and was accepted as an indicator of circulating platelets in a unit volume of blood [19, 20].

The available data regarding the relationships between antiplatelet drugs and large platelets are lacking. Particularly, aspirin does not seem to significantly affect platelet volume, either in vitro or in vivo [21]*.* Conversely, large platelets may have an increased reactivity even in patients with stable coronary artery disease undergoing dual antiplatelet therapy with aspirin and clopidogrel [22]*.*

The goal of this study is to determine out whether an association exists between MPV and PCT and outcome of acute ischemic stroke.

## Patients and methods

This prospective study includes 100 and 57 consecutive patients (36.9% males and 63.1% females) with acute ischemic stroke, admitted from August 2013 to October 2014 to the Neurology Department, Sohag University Hospital.

We obtained an informed consent and written when possible from the patients or relative. The study was approved by the local ethical committee of Faculty of Medicine, Sohag University in April 2013.

These patients were attended for first-ever acute ischemic stroke, which is defined as focal neurological signs or symptoms thought to be of vascular origin that persisted for > 24 h [23] confirmed by brain computed tomography (CT) and/or MRI. Those with clinical data corresponding to stroke with normal brain CT scan result were also considered as ischemic stroke, but another CT scan was done after 2 days to measure the maximum lesion diameter. The cerebral lesions whose maximum diameter was ≥ 4 cm were considered large infarction [12].

We exclude patients with transient symptoms of cerebrovascular diseases, peripheral vascular disease, acute infection, positive C-reactive protein or inflammatory conditions, prior stroke, acute myocardial infarction, malignancies, and intracranial hemorrhage. Also, patients who administered medications including anti-lipids, angiotensin-converting enzyme inhibitors, and anticoagulants were also excluded.

No patient underwent IV or IA thrombolysis, during the study period, but the participants received the usual antiplatelet treatment.

Full medical and neurological evaluation was done for all patients, including demographics; vascular risk profile; medical history, especially the former history of ischemic heart disease or stroke; biochemical parameters; medications that may affect platelet count; echocardiographic data; and neuroimaging studies. Canadian stroke scale (CSS) was done to determine stroke severity with a score of 6.5 on CSS as a cut point to differentiate a more and less severe stroke, and lower scores indicate greater stroke severity [24, 25].

Glasgow coma scale was used to assess conscious level of the patient and was graded as mildly disturbed (GCS = 13–15), moderately disturbed (GCS = 9–12), and severely disturbed (GCS ≤ 8) [26].

For the assessment of blood pressure, high blood pressure was defined as the use of anti-hypertensive drugs or persistently elevated blood pressure (> 140/90 mmHg) on admission.

Diabetes mellitus was defined as the use of hypoglycemic agents or a fasting plasma glucose of > 126 mg/dl (after no caloric intake for at least 8 h) or casual plasma glucose > 200 mg/dl [27].

All patients underwent blood sampling immediately after admission to the Neurology Department and before administration of medication or intravenous fluid. A complete blood count was performed, and the platelet parameters obtained. The samples were collected in EDTA tubes and transported to the laboratory. Platelet measurements were performed within 2 h to ensure minimal platelet swelling [28, 29] by a Cell Dyn 3700 hematology analyzer that provided platelet count and MPV (in fL). PCT (expressed in percentage) is determined by multiplying the platelet number in thousands by mean platelet volume in femtoliters and dividing that product by 10,000. PCT was determined by using the same equation as the Cell Dyn 3700 hematology analyzer: PCT = (PLT × MPV)/10,000 [10].

Typically, the average mean platelet volume is 7.2–11.7 fL in healthy subjects while the normal range for PCT is 0.22–0.24% [30, 31].

Other routine laboratory investigations were done like liver function tests, renal function tests, and blood sugar test. Lipogram was done; cholesterol level was considered normal when total cholesterol is less than 200 mg/dL; LDL (“bad” cholesterol) level is less than 100 mg/dL, while HDL (“good” cholesterol) level is 60 mg/dL or higher; and normal triglycerides is less than 150 mg/dL [32].

The outcome of ischemic stroke was assessed after 3 months of the insult by modified Rankin Scale (mRS) that scores patients on a scale from 0 to 6, with 0 being asymptomatic and 6 being dead. Scores of 0–2 are considered “favorable” stroke outcomes while scores of 3 or greater indicate “unfavorable” outcomes [33, 34].

### Statistical analyses

The data were analyzed by Statistical Package for the Social Sciences (SPSS 20.0, IBM Corp., Armonk, NY, USA).

The groups with the different ischemic stroke outcome were compared using the Student’s *t* test for the continuous variables and the chi-square test for the categorical variables. Predictors exhibiting a statistically significant relation with poor outcome (mRS ≥ 3) were taken for multivariate logistic regression analysis to investigate their independence as predictors. The adjusted odds ratio (OR) and 95% confidence intervals (CI) were calculated. Statistical significance was determined at *P* ≤ 0.05.

## Results

A total of 157 patients with first-ever ischemic stroke with a mean age of 56.7 ± 16.2 years, and 36.9% males and 63.1% females were included to this study (Table 1).

**Table 1:** Baseline characteristics of patients

	Number	Percent
Age (mean ± SD)	56.72 ± 16.218	
Sex		
Male	58	(36.9)
Female	99	(63.1)
Smoking		
Nonsmoker	129	(82.2)
Smoker	28	(17.8)
Hypertensive	67	(42.7)
SBP (mean ± SD)	144.8 ± 27.0	
DBP (mean ± SD)	94.7 ± 22.43	
DM	50	(31.8)
Blood glucose level (mean ± SD)	129.6 ± 63.2	
Cardiac	33	(21.0)
Platelet indices		
MPV (mean ± SD)	10.3 ± 2.6	
PCT (mean ± SD)	0.26 ± 0.11	
Lipogram		
Total cholesterol (mean ± SD)	183.4 ± 46.3	
Triglycerides (mean ± SD)	131.1 ± 66.04	
LDL (mean ± SD)	114.6 ± 45.05	
HDL (mean ± SD)	39.1 ± 10.4	
Lesion size (CT and/or MRI)		
Less than 4 cm	134	(85.4)
≥ 4 cm	23	(14.6)
Conscious level measured by GCS)		
Canadian stroke		
Mild to moderate stroke	86	54.8
Severe stroke	71	45.2
Mildly disturbed (13–15)	107	68.2
Moderately disturbed (9–12)	48	30.6
Severely disturbed (≤ 8)	2	1.3
Outcome (measured by mRS)		
Favorable outcome	81	51.6
Unfavorable outcome > 3	76	48.4

*SBP* systolic blood pressure, *DBP* diastolic blood pressure, *DM* diabetes mellitus, *MPV* mean platelet volume, *PCT* plateletcrit, *LDL* low-density lipoprotein, *HDL* high-density lipoprotein, *CT* computerized tomography, *MRI* magnetic resonance imaging, *GCS* Glasgow coma scale, *mRS* modified Rankin scale

The favorable outcome was 51.6%, whereas the unfavorable one was 48.4%. Table 1 and Table 2 summarize the main characteristics of the patients in both groups. Age was significantly correlated with patients of the unfavorable group (*P* value < 0.001). Patients with favorable outcome were less likely to have a history of hypertension and cardiac diseases. Mean platelet volume and PCT were significantly higher in patients with unfavorable outcome with *P* value < 0.001 and *P* value = 0.041 respectively. Table 3 shows multivariate logistic regression analysis and odds ratios for each of the factors. The significant factors in the model were age (*P* value = *0.001*), MPV (*P* value **<** *0.001*), DM (*P* value = *0.046*), and triglycerides (*P* value = *0.008*) which were considered as independent predictors for stroke outcome after controlling for confounders like age, DM, and triglycerides.

**Table 2:** Characteristic of patients in both favorable and unfavorable groups

	Favorable outcome MRS ≤ 2 (*N* = 81)	Unfavorable outcome MRS > 3 (*N* = 76)	*P* value
Age	51 ± 17.8	62.7 ± 11.6	< 0.001
Sex			0.524
Male	28 (34.6%)	30 (39.5%)	
Female	53 (65.4%)	46 (60.5%)	
Smoking	12 (14.8%)	16 (21.1%)	0.308
Cardiac disease	10 (12.3%)	23 (30.3%)	0.006
Hypertensive	22 (27.2%)	45 (59.2%)	< 0.001
Diabetic	12 (14.8%)	26 (34.2%)	0.005
GCS	14.3 ± 1.3	12.62 ± 2.3	< 0.001
Platelet measurements			
MPV	8.7 ± 1.3	10.4 ± 2.3	< 0.001
Plateletcrit (PCT)	0.25 ± 0.10	0.28 ± 0.12	0.041
Canadian stroke scale	8.1 ± 2.3	4.6 ± 3.2	< 0.001

*mRS* modified Rankin scale, *GCS* Glasgow coma scale

**Table 3:** Predictors of stroke outcome by using multivariate logistic regression analysis for determining the role of MPV and PCT in predicting stroke outcome

Characteristics	Regression coefficients *β*	SE	Odds ratio	95% C.I.		*P* value
				Lower	Upper	
Age	0.060	0.017	1.062	1.027	1.099	0.001
DM	1.036	0.520	2.817	1.016	7.809	0.046
MPV	0.432	0.105	1.540	1.253	1.893	< 0.001
PCT	0.430	2.359	1.537	0.015	156.369	0.855
Cholesterol	0.007	0.006	1.007	0.996	1.019	0.207
Triglycerides	− 0.010	0.004	0.990	0.982	.997	0.008
Constant	− 8.359	1.973	0.000		1.099	

*MPV* mean platelet volume, *PCT* plateletcrit*, SE* standard error

## Discussion

Increased platelet volume may interpret the net patho-physiological effects of a number of cerebrovascular diseases (CVD) risk factors, such as smoking and elevated cholesterol, therefore presenting a broad marker of CVD risk. Several studies reported that high MPV levels is associated with overall vascular mortality in the general population [35–38]. In contrast, some studies did not reveal any relationship between platelet volume and function with the outcome of ischemic stroke which may be explained by time-dependent platelet swelling in vitro and the influence of MPV by various comorbidities and concomitant drug therapies like antiplatelet and perindopril [9, 39, 40]. There is a current growing evidence that platelet indices including MPV and PCT are significantly correlated with vascular risk factors and can predict prognosis of acute ischemic stroke [2–4, 41].

The result of this work demonstrated that increased MPV is associated with poor functional outcome of stroke and highlighted the importance of MPV as an independent predictor for stroke outcome after controlling for confounders like DM which is confirmed by previous studies [11].

Previous studies reported that higher MPV was associated with the larger infarct size and higher risk of post-stroke mortality [12, 42].

On the contrary, two studies [39, 43] did not find any significant relation between MPV and stroke outcome and the causes for these inconsistent findings could be explained by small numbers of patients and the use of different methodological measures of platelet indices and different outcome scales in these studies [28].

The studies conducted to detect the relationship between PCT and stroke outcome are lacking, and the results were conflicting; one earlier study found no difference in platelet mass in stroke patients compared to control [9] while the other found a decrease in platelet count, MPV, and plateletcrit in patients with lacunar infarction [10].

Like previous studies, the present work confirms that increased MPV and subsequent higher platelet mass (plateletcrit) are significantly correlated with short-term outcome of ischemic stroke [11, 28, 41] but not considered as independent predictor, and further studies are warranted to explain this correlation.

The mechanisms by which elevated MPV might play a role in the development or progression of cardiovascular diseases are not completely understood, but there are some possible explanations. First, platelet reactivity is increased with ischemic stroke as evidenced by the increased levels of soluble platelet P-selectin and increased levels of thromboxane A2 which are considered as atherogenic factors [44, 45]. Second, cytokines such as interleukin-3 or interleukin-6 which plays an important role in the pathophysiology of ischemic stroke influence megakaryocyte ploidy which in turn affect the platelet size and can lead to the production of more reactive, larger platelets [28] and so the proinflammatory condition before the ischemic stroke may lead to a higher MPV which in turn lead to prothrombotic condition. Finally, large platelets may be a consequence of secretion and metabolism of biologically active substances during aging, diabetes, high blood pressure, and obesity and consequently cause increased risk of ischemic stroke [41, 46, 47].

### Limitations

This study has some limitations. One is changeability of platelet size because of time-dependent platelet swelling in vitro using EDTA as an anticoagulant. Second, serial MPV measurements were not done during different stages of stroke and so variability of platelet size during stroke evolutions could not be estimated.

## Conclusion

The present study exhibits an association between MPV and PCT and acute ischemic stroke functional outcome. These findings raise the potential importance of MPV and PCT as easily obtained during routine hematologic analysis, as a valuable prognostic tool for outcome of cerebrovascular stroke. Further research is required to accurately determine the role of platelet volume in stroke pathology, outcome, and, most importantly, in individuals at risk for stroke.

## Abbreviation

CSS: Canadian stroke scale
CVD: Cerebrovascular diseases
MI: Myocardial infarction
MPV: Mean platelet volume
mRS: Modified Rankin Scale
PCT: Plateletcrit
